# Delayed Small Bowel Incarceration Within a Lumbar Burst Fracture After Posterior Spinal Fusion: A Case Report

**DOI:** 10.7759/cureus.4708

**Published:** 2019-05-21

**Authors:** Elliot Min, Michael F Barbaro, Joseph Chen, Charles Liu, Brian Lee

**Affiliations:** 1 Neurosurgery, University of Southern California, Los Angeles, USA

**Keywords:** small bowel obstruction, lumbar burst fracture, posterior spinal fusion

## Abstract

We present a case report of a patient who had delayed small bowel obstruction secondary to an incarcerated loop of small bowel within an acute lumbar spine fracture. The patient was involved in a rollover motor vehicle accident, resulting in lumbar spine fractures at L2-4. A comminuted fracture of the L3 vertebral body with likely disruption of the anterior longitudinal ligament (ALL) was noted. The patient underwent L1-4 posterior spinal fusion with the introduction of mild lumbar lordosis to prevent future complications of flatback syndrome. On postoperative Day 4, the patient was noted to have signs and symptoms of progressive small bowel obstruction. Conservative management was initiated with minimal improvement. The patient was taken to the operating room on postoperative Day 7 for an exploratory laparotomy. A necrotic loop of small bowel was noted to be entrapped in the ventral L3 vertebral body defect. This bowel was released and resected, with side-to-side anastomosis performed. No previous cases describe small bowel incarceration because of posterior spinal fusion for trauma. The introduction of increased lumbar lordosis is thought to have contributed to this risk of small bowel herniation, and care must be taken when determining appropriate spinopelvic parameters in these cases.

## Introduction

Bowel obstruction secondary to entrapment within the lumbar spine after trauma has been previously described in the literature but is exceedingly rare [1-12]. Here, we present the case report of a patient with an L2-4 fracture after a motor vehicle accident complicated by a delayed small bowel obstruction (SBO) from incarceration within the L3 vertebral body resulting in necrosis.

## Case presentation

A 28-year-old man presented to the emergency department after a motor vehicle accident. He was the restrained driver of an automobile traveling at 50 miles per hour when he was struck by another vehicle, rolled over, and was ejected from his car. He was assisted to the standing position by emergency services but was unable to ambulate due to pain. His main complaint was back pain, which he reported was similar to the pain he experienced from a prior compression fracture.

**Figure 1:**
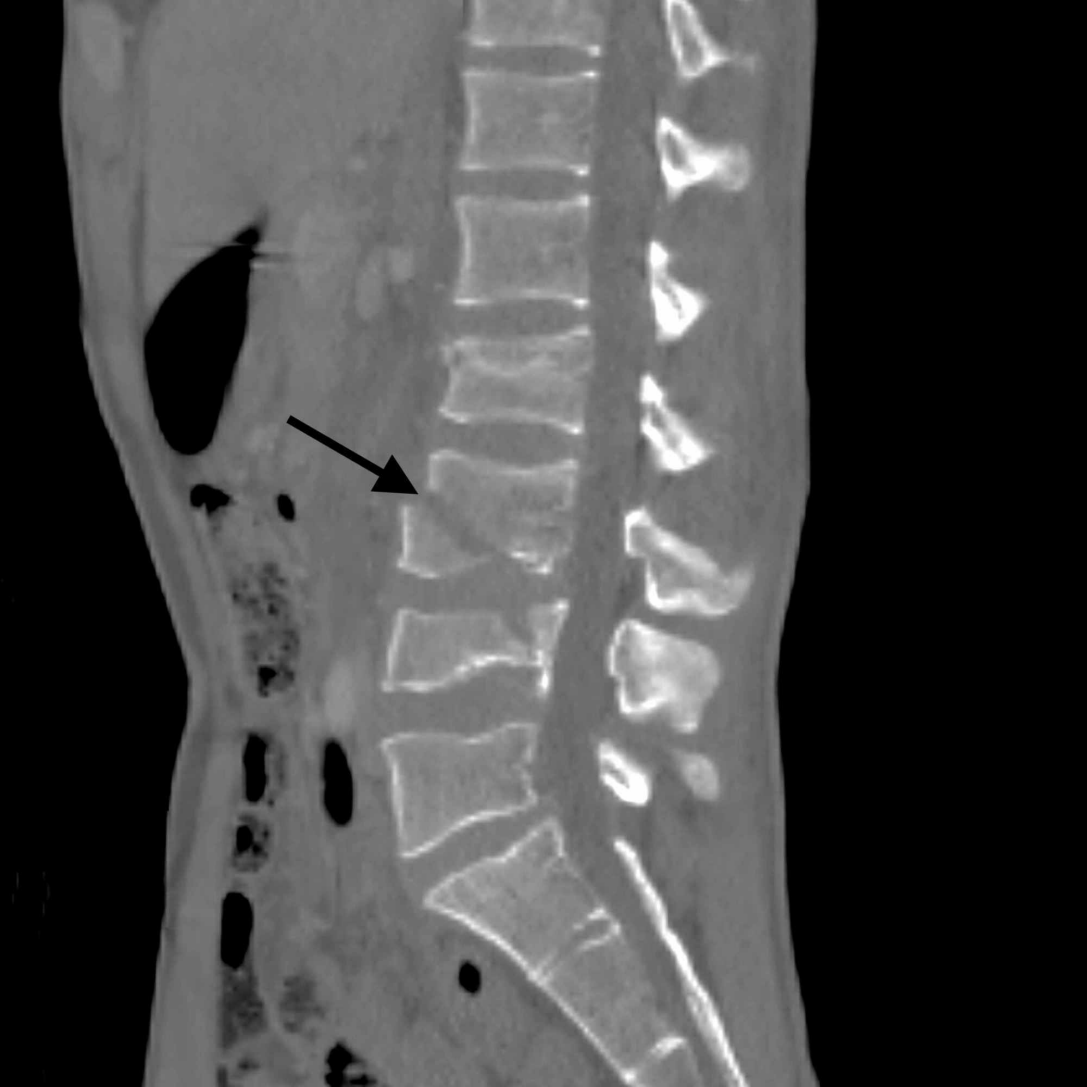
Sagittal CT demonstrating L2-4 fractures. Note the comminution of L3 and L4 resulting in a ventral defect of L3.

**Figure 2:**
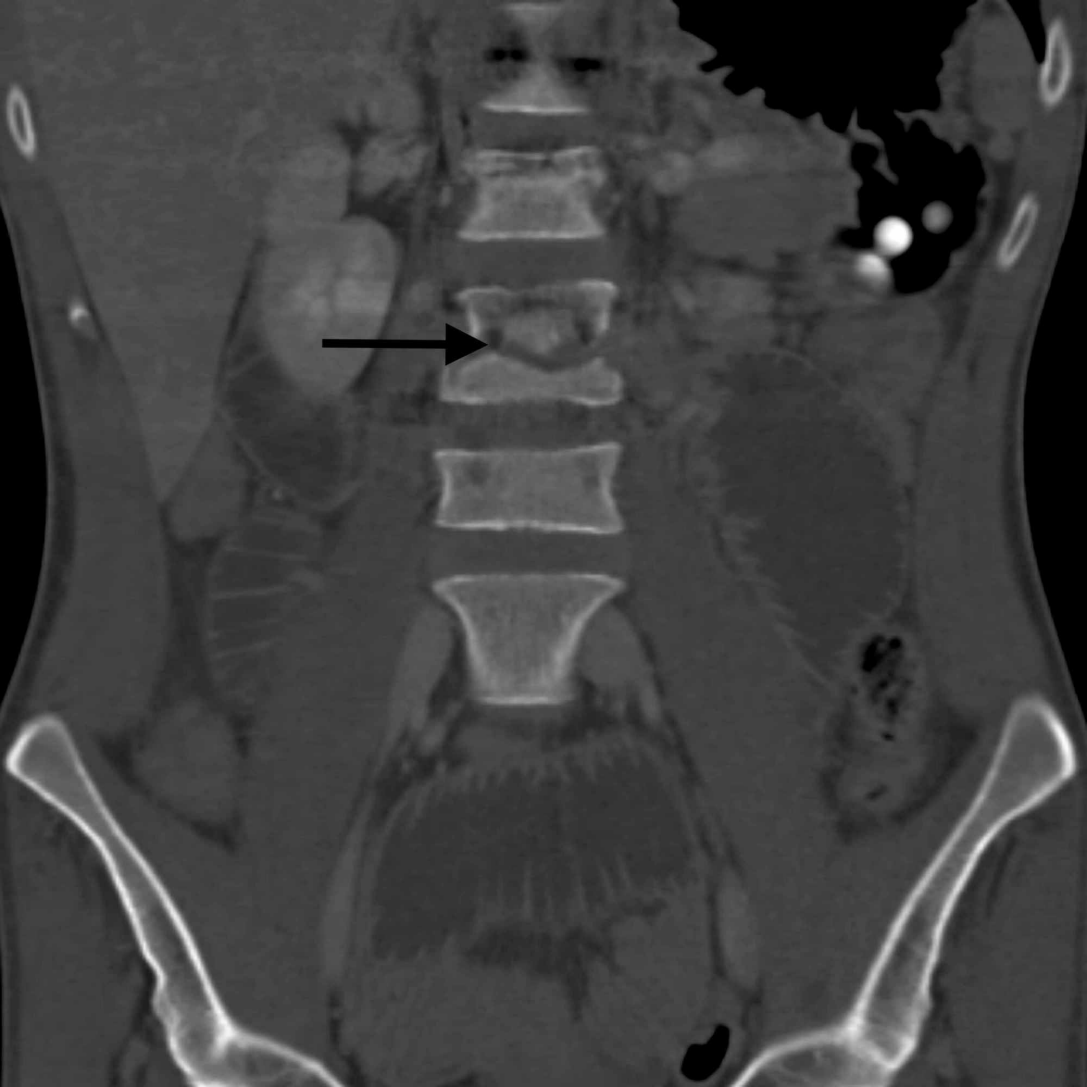
Coronal CT showing a ventral defect at L3.

**Figure 3:**
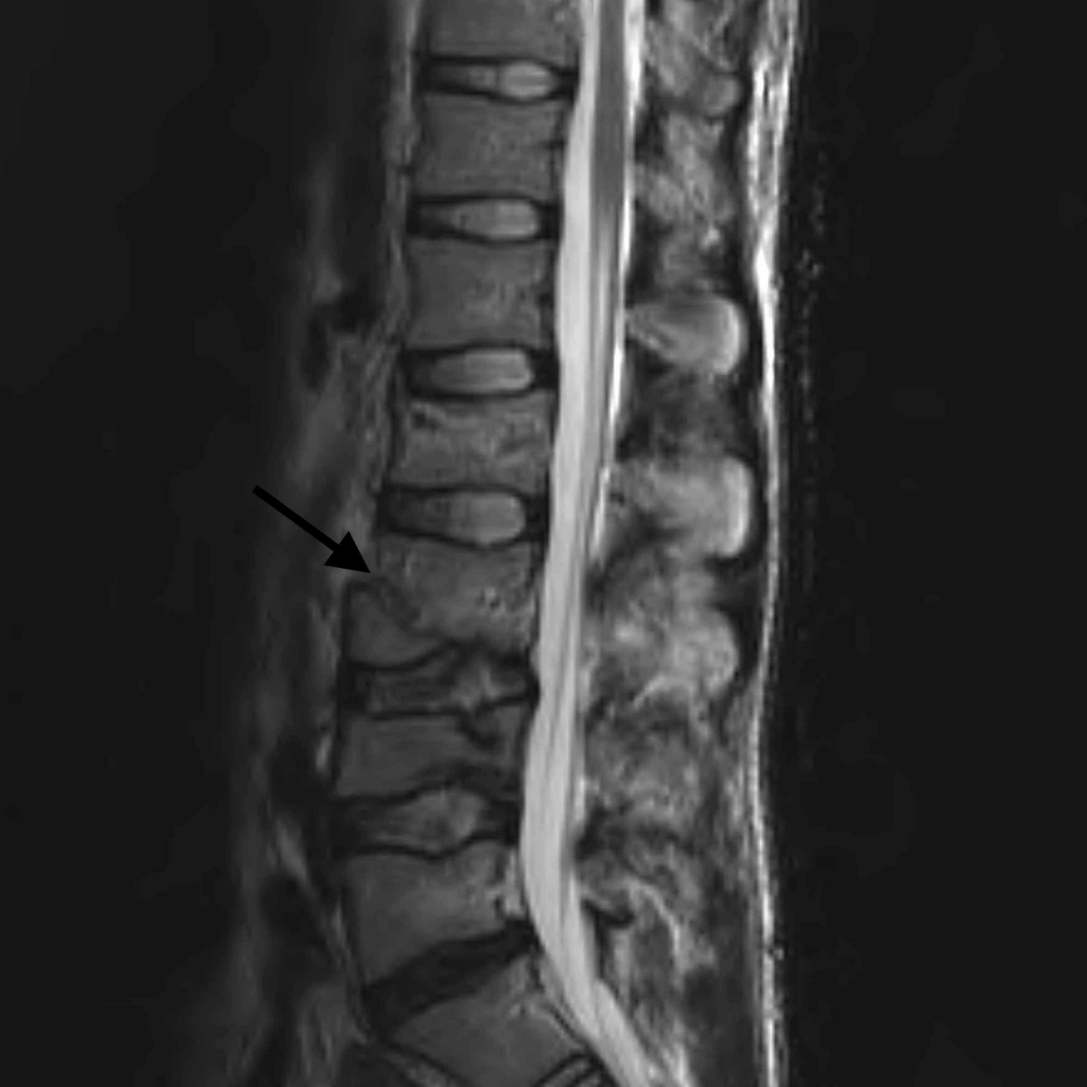
Sagittal T2 MRI showing disruption of the anterior longitudinal ligament (ALL).

**Figure 4:**
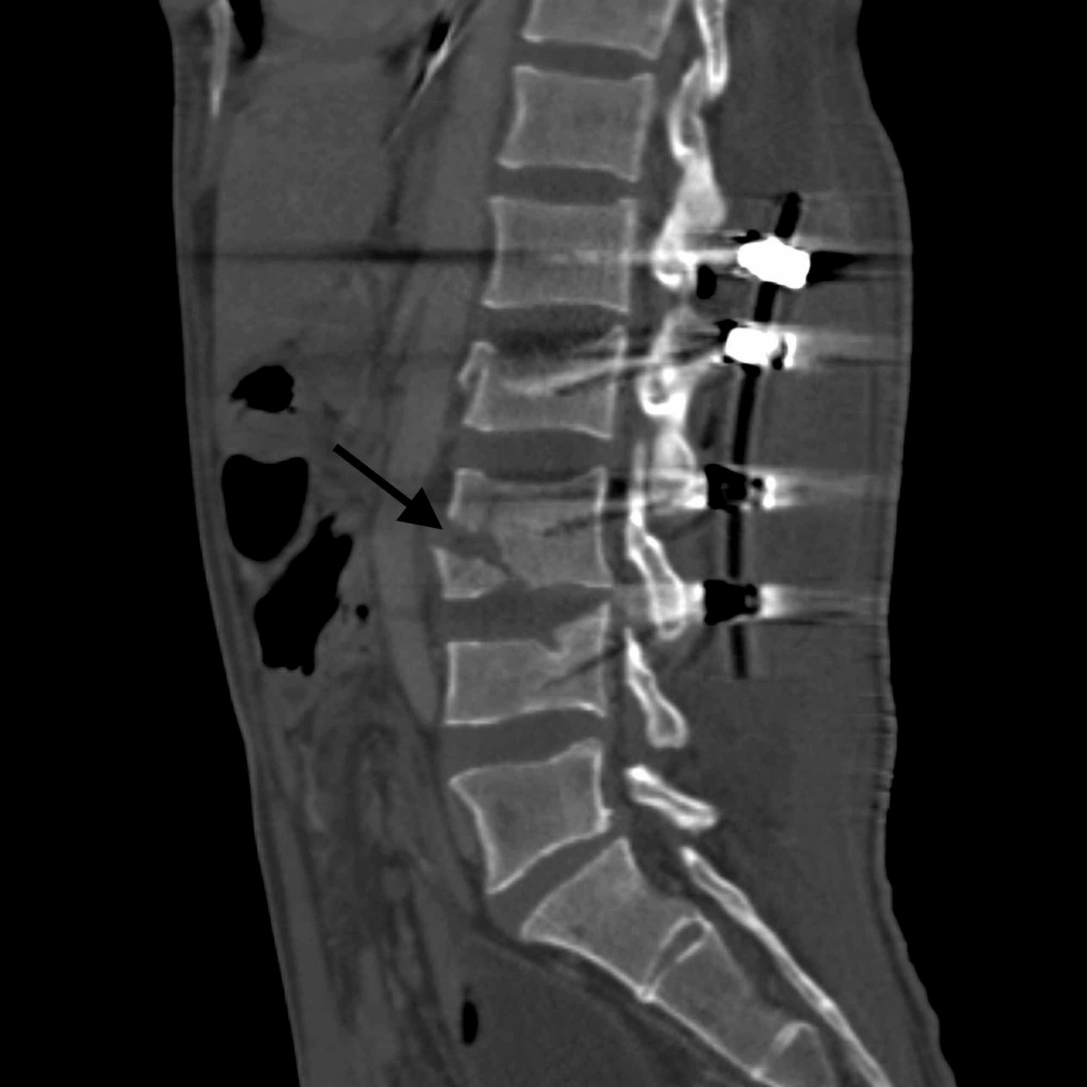
Postoperative sagittal CT demonstrating a slight widening of the ventral defect at L3.

Upon arrival to the emergency room, he was noted to have cervical and lumbosacral tenderness to palpation without significant step-off deformity on physical exam. He denied neurological symptoms and was neurologically intact on examination. He had no symptoms of abdominal pain, distention, or tenderness to palpation at that time. A computed tomography (CT) scan of the abdomen and pelvis was performed, demonstrating an acute fracture from L2-L4 with mild retropulsion of the posterior L3 and L4 vertebral bodies into the spinal canal (Figures 1-2). Magnetic resonance imaging (MRI) of the lumbar spine was performed, which showed a possible injury of the posterior ligamentous complex (Figure 3). According to the Thoraco-Lumbar Injury Classification and Severity Score (TLICS), the patient had a TLICS score of 4. According to the AOSpine thoracolumbar spine injury classification system, the patient had an injury consistent with type A2. Given the significant deformity, the decision was made to perform a posterior spinal fusion from L1-L4. Pedicle screws were placed from L1-L4 in standard fashion, and rods were bent to provide mild lordosis to prevent flatback syndrome (Figure 4). The case was completed without complication and the patient was transferred to the floor for postoperative observation.

**Figure 5:**
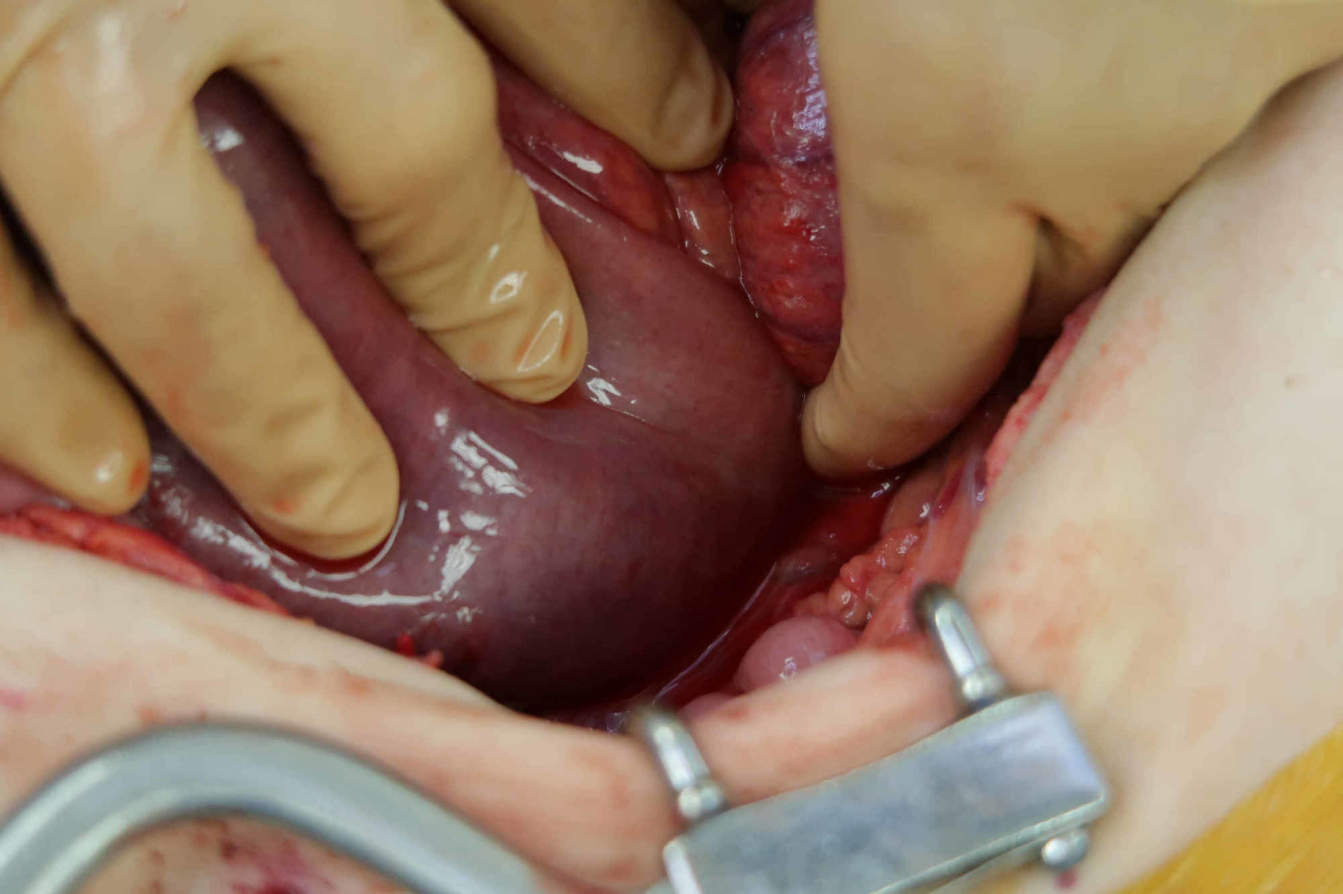
Intraoperative photo demonstrating the small bowel tethered to the ventral lumbar spine.

**Figure 6:**
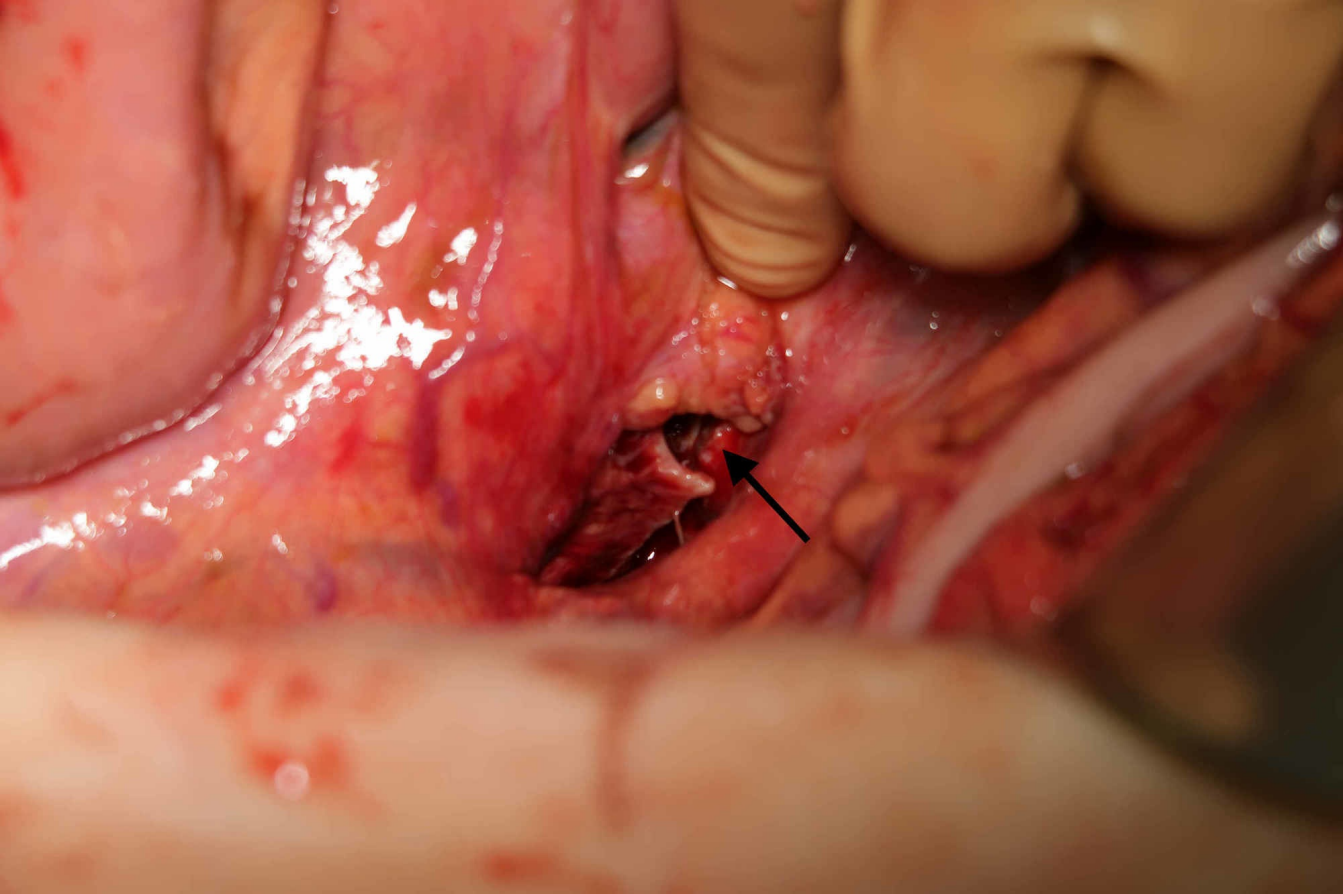
Ventral vertebral body defect after the entrapped bowel was released.

**Figure 7:**
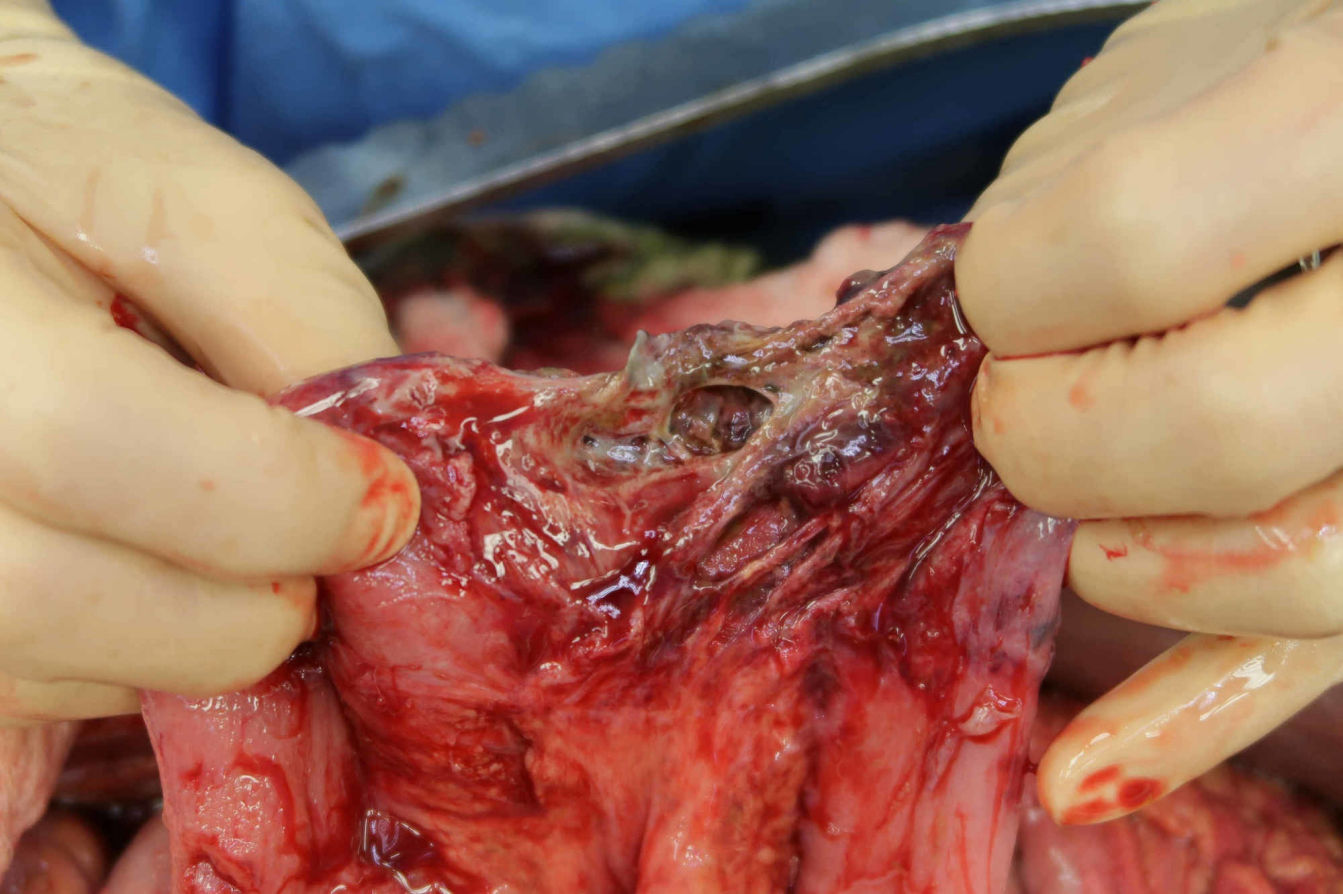
A segment of necrotic small bowel released from vertebral entrapment.

**Figure 8:**
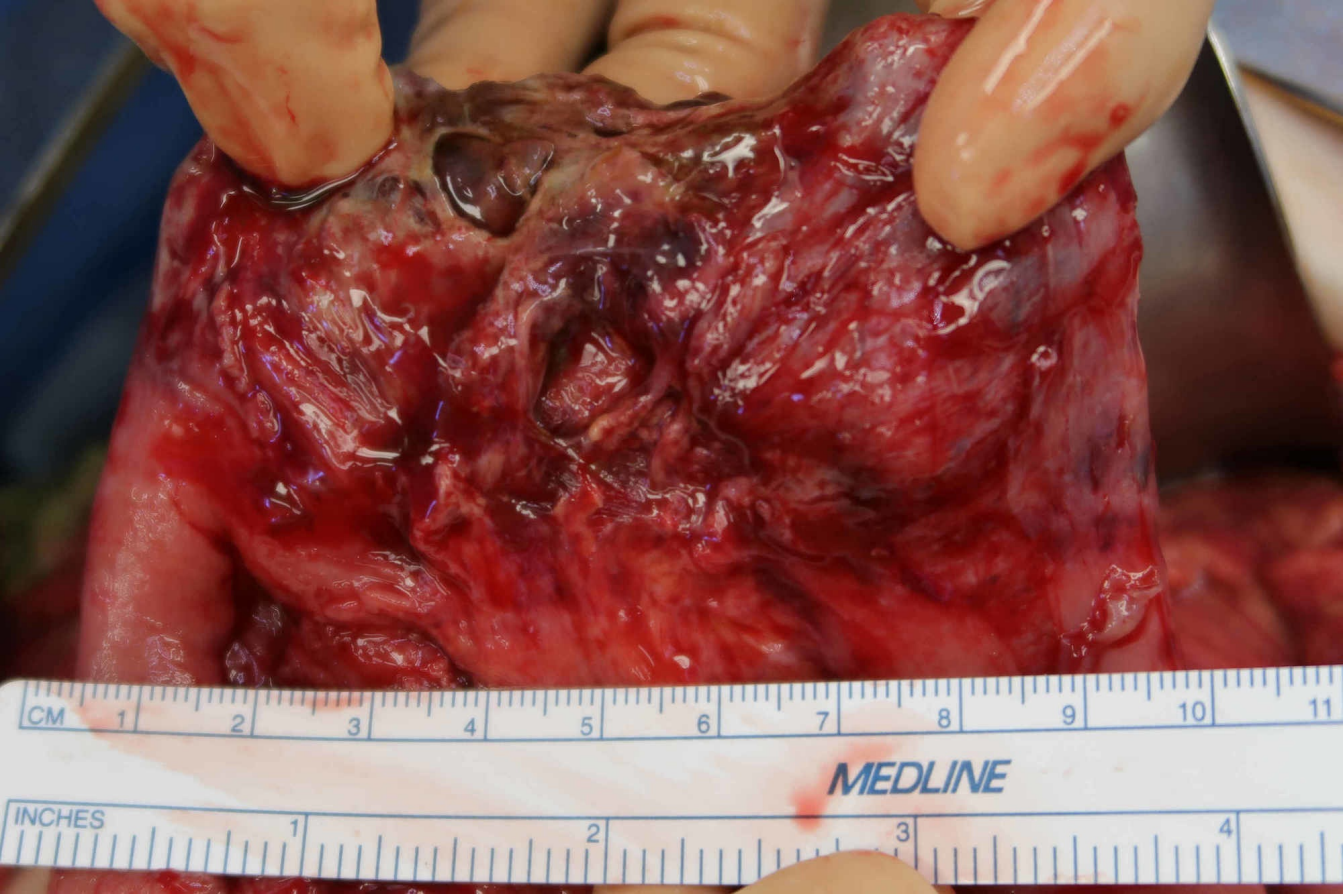
Nine cm of small bowel noted to be necrotic and resected.

**Figure 9:**
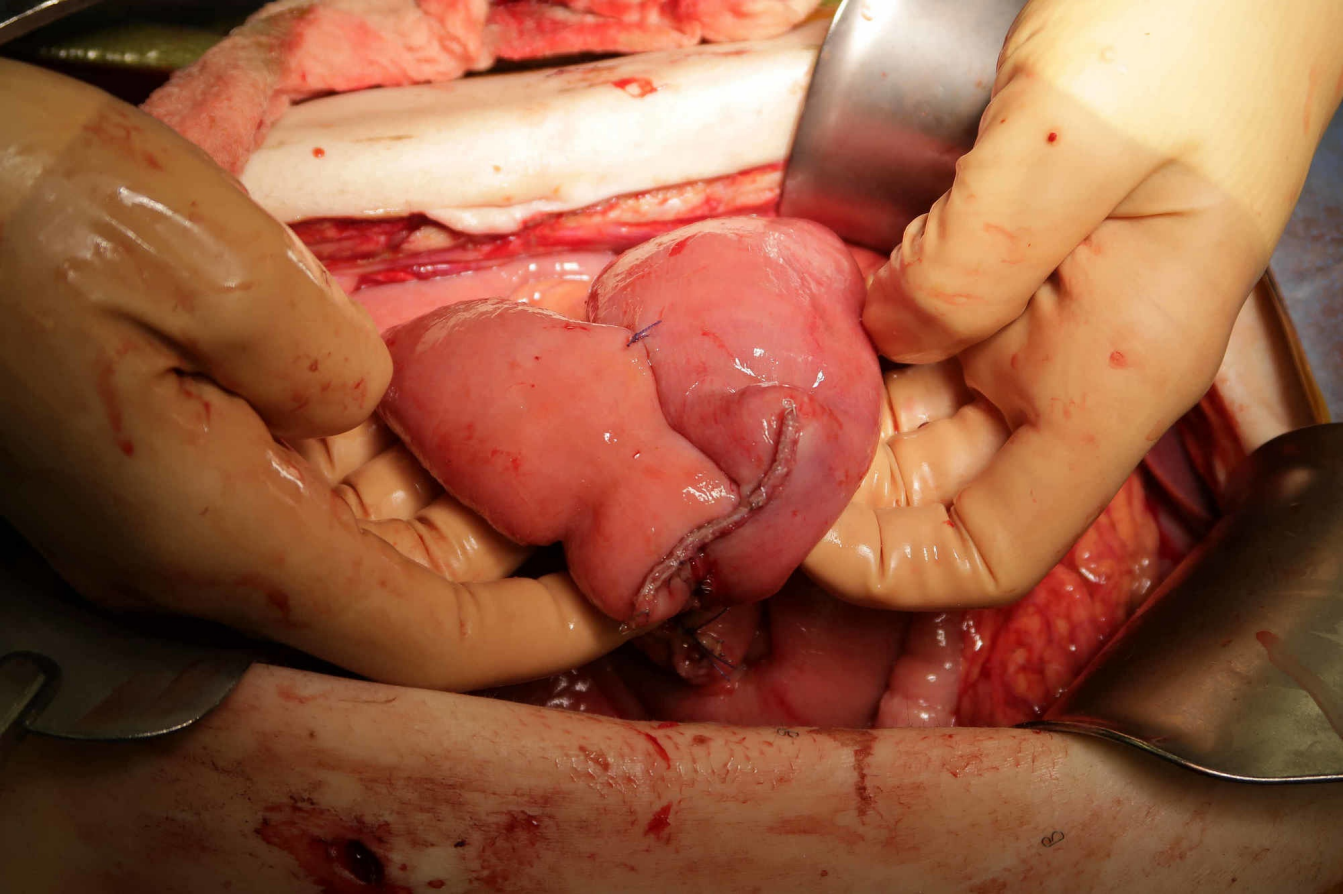
After bowel resection, primary side-to-side anastomosis of the bowel was performed and the bony defect was packed with bone wax to prevent further herniation.

On postoperative Day 4, the patient developed acute onset nausea and vomiting. Prior to this, the patient was noted to be passing gas but had not had a bowel movement. An abdominal radiograph was obtained demonstrating distended loops of bowel. An abdominal CT was obtained demonstrating a dilated bowel but no evidence of free air. The patient was made NPO, a nasogastric tube was placed, and he was started on supportive therapy for presumed small bowel obstruction. The patient continued to experience vomiting and on postoperative Day 7, he underwent exploratory laparotomy for small bowel obstruction. Intraoperatively, a 9 cm loop of bowel was noted to be incarcerated and strangulated in the center of the L3 vertebral body at the fracture site (Figures 5-6). This necrotic bowel was released from the defect in the vertebra, which was packed with bone wax. Bowel resection and side-to-side anastomosis were then performed (Figures 7-9).

The patient was started on broad-spectrum antibiotics postoperatively. He was ultimately discharged home with a lumbosacral orthosis and home health antibiotics on postoperative Day 18.

## Discussion

SBO secondary to entrapment within a lumbar spine fracture has been previously described but is noted to be exceedingly rare [1-12]. We present a case of delayed intestinal obstruction as a result of incarceration within a lumbar spine fracture after lumbar posterior spinal fusion. To our knowledge, this is the first documented case of bowel incarceration within a lumbar burst fracture; prior cases describe bowel herniation into the intervertebral space secondary to traumatic hyperextension or lumbar spondylolisthesis. Additionally, our case is the first to report delayed obstruction diagnosed after lumbar posterior spinal fusion.

A potential space in the lumbar spine through which abdominal contents can herniate is thought to result from a hyperextension injury with rupture of the anterior longitudinal ligament (ALL) or disc space between vertebral bodies or from a sudden axial load resulting in a fracture of the vertebral body itself through the ALL. Two theories are proposed to explain bowel incarceration within this bony defect. First is that an anterior force (i.e. from a seatbelt) pushes the bowel posteriorly. The other is that the generation of this potential space generates a vacuum effect that entraps the bowel [7]. In this particular case, the patient had a defect within the vertebral body itself. Before and immediately after undergoing a lumbar posterior spinal fusion, the patient had no clinical signs or symptoms of SBO. Additional lumbar lordosis was introduced during rod placement, which may have helped to create a larger ventral vertebral body defect through which bowel contents could enter. Postoperative imaging demonstrated that the original defect was widened after posterior spinal fusion.

Incarceration of any intraperitoneal/retroperitoneal contents can theoretically occur in the setting of a spinal defect. Defects in the ALL and the vertebral column (i.e. vertebral body and disc) can occur from trauma or infection. Kim et al*.* report a case of osteodiscitis resulting in damage to the intervertebral disc and ALL, leading to invagination of the inferior vena cava [13]. The most commonly reported structure to be entrapped is the jejunum, as a result of acute trauma [1-6,8,10-12]. Besides small bowel segments, other structures have been shown to herniate into a widened ventral space, including common iliac vessels [14], the inferior vena cava [13,15], retroperitoneal fat, and the psoas muscle [13]. Pesenti et al. present a case involving an L3 chance fracture resulting in small bowel herniation within the spinal canal, resulting in cauda equina syndrome [8]. Another case from Ko et al. describes the entrapment of the sigmoid colon within the L5-S1 interspace, causing S1 nerve root compression and radiculopathy requiring laparotomy, and the reduction of the colon combined with anterior lumbar interbody fusion [16].

The diagnosis of traumatic incarceration of the small bowel often occurs in a delayed fashion, resulting in potentially increased morbidity secondary to abdominal complications. The primary limitation of diagnosing an associated small bowel obstruction is that the vertebral injury itself often masks associated symptoms. Abdominal pain is often concealed or overlooked as a result of pain from the spine fracture. Ileus can also result from vertebral fractures or retroperitoneal hematomas and is more commonly seen than SBO. Symptoms such as nausea and vomiting are generally treated conservatively, with supportive care, which can lead to further necrosis and sepsis from bowel incarceration [1]. Abdominal radiographs can potentially diagnose small bowel obstruction but lack the diagnostic capability of a CT scan or MRI. Patients with abdominal symptoms concerning for small bowel obstruction in the setting of a lumbar spine fracture should undergo further imaging to evaluate for possible bowel incarceration. However, the CT scan may not demonstrate the actual herniation itself. Davis et al. describe a case of small bowel entrapment where CT only demonstrated retroperitoneal free air and thickened bowel at the level of L2, without imaging evidence of bowel herniation [1]. Ultimately, exploratory laparotomy and direct visualization are necessary to make the diagnosis.

Regarding surgical management, introducing more lumbar lordosis during fusion may have further extended the fracture site, resulting in a vacuum phenomenon and a larger opening through which abdominal contents could herniate. While restoring lumbar lordosis in the setting of a vertebral fracture is important in maintaining sagittal balance and preventing flatback syndrome, care must be taken when a large ventral defect is noted preoperatively. Recognizing this scenario may decrease the risk of subsequent bowel entrapment.

## Conclusions

While uncommon, small bowel incarceration should be considered in lumbar spine trauma patients with abdominal complaints. Hyperextension injuries or burst fractures resulting in traumatic disruption of the ALL and vertebral body should increase this suspicion. Although further imaging may be useful in the assessment of these patients, exploratory laparotomy is often required for diagnosis. Surgical treatment of such fractures has the potential to increase the risk of bowel entrapment via the introduction of a vacuum phenomenon or an increase in the size of the vertebral defect via increased lumbar lordosis. This risk should be recognized when determining appropriate sagittal balance and spinopelvic parameters.
